# Performance Evaluation of Different Object Detection Models for the Segmentation of Optical Cups and Discs

**DOI:** 10.3390/diagnostics12123031

**Published:** 2022-12-02

**Authors:** Gendry Alfonso-Francia, Jesus Carlos Pedraza-Ortega, Mariana Badillo-Fernández, Manuel Toledano-Ayala, Marco Antonio Aceves-Fernandez, Juvenal Rodriguez-Resendiz, Seok-Bum Ko, Saul Tovar-Arriaga

**Affiliations:** 1 Faculty of Engineering, Autonomous University of Querétaro, Santiago de Querétaro 76010, Mexico; 2 Department of Electrical and Computer Engineering, University of Saskatchewan, 57 Campus Drive, Saskatoon, SK S7N 5A9, Canada; 3 Instituto Mexicano de Oftalmología (IMO) I.A.P., Circuito Exterior Estadio Corregidora sn, Centro Sur, Santiago de Querétaro 76010, Mexico

**Keywords:** glaucoma, segmentation, object detection, Mask R-CNN, Cascade Mask R-CNN, instance segmentation, average precision, intersection over union

## Abstract

Glaucoma is an eye disease that gradually deteriorates vision. Much research focuses on extracting information from the optic disc and optic cup, the structure used for measuring the cup-to-disc ratio. These structures are commonly segmented with deeplearning techniques, primarily using Encoder–Decoder models, which are hard to train and time-consuming. Object detection models using convolutional neural networks can extract features from fundus retinal images with good precision. However, the superiority of one model over another for a specific task is still being determined. The main goal of our approach is to compare object detection model performance to automate segment cups and discs on fundus images. This study brings the novelty of seeing the behavior of different object detection models in the detection and segmentation of the disc and the optical cup (Mask R-CNN, MS R-CNN, CARAFE, Cascade Mask R-CNN, GCNet, SOLO, Point_Rend), evaluated on Retinal Fundus Images for Glaucoma Analysis (REFUGE), and G1020 datasets. Reported metrics were Average Precision (AP), F1-score, IoU, and AUCPR. Several models achieved the highest AP with a perfect 1.000 when the threshold for IoU was set up at 0.50 on REFUGE, and the lowest was Cascade Mask R-CNN with an AP of 0.997. On the G1020 dataset, the best model was Point_Rend with an AP of 0.956, and the worst was SOLO with 0.906. It was concluded that the methods reviewed achieved excellent performance with high precision and recall values, showing efficiency and effectiveness. The problem of how many images are needed was addressed with an initial value of 100, with excellent results. Data augmentation, multi-scale handling, and anchor box size brought improvements. The capability to translate knowledge from one database to another shows promising results too.

## 1. Introduction

Near 2.2 billion people have a vision disability or blindness, and one billion have a condition that could have been prevented or has not yet been treated [1].

Glaucoma is a common cause of irreversible blindness, and it is associated with essential pathophysiology such as the retinal ganglion cell (RGC), stroma, photoreceptors, lateral geniculate body, and visual cortex. However, RGC axons lost inside of the optic nerve head (ONH) are the leading cause of vision loss. Because of that, glaucoma is considered a multifactorial disease, influenced at least by intraocular pressure (IOP), blow flow and ischemia inside laminar and prelaminar tissues, and an autoimmune or inflammatory state of the tissues [2]. Regarding prognosis, most of the time, the illness can be controlled with early diagnosis and adequate medication and care.

Diagnosing the presence of the disease requires expertise. It involves interpreting several specialized tests, such as IOP, visual field, and assessment of damaged ONH, through 3D imaging techniques such as Heidelberg Retinal Tomography (HRT) and Ocular Computing Tomography (OCT) [3]. However, these evaluation methods are often hampered by the high cost of some medical equipment unavailable in many hospitals. Visual-field analysis often depends on the patient’s cooperation.

Important features that can be taken from retinal images are the following: cup-to-disc ratio (CDR), which, for a standard disc, should be less than 0.3, as higher is considered possible glaucoma; neuro-retinal rim, which is the area between the edges of the disc and the optical cup; and inferior-superior-nasal-temporal rule (ISNT), in which inferior must be higher than superior, and superior higher than nasal, and nasal higher than temporal to accomplish the rule [4].

For this reason, a considerable amount of research proposes a detection and segmentation of the ONH to extract insights from it. The ONH can be found in the retina, the layer of nerve cells lining the back wall of the eye. This layer detects light and sends signals for image formation to the brain through elements called cones and rods [5].

An extensive review of literature can be found in [6], with methods such as Level Set-based, threshold-based, clustering-based, and artificial-neural-network, the last one with more general precision and less processing time but high computational cost. A recent review paper [7] summarizes existing public datasets as well as machine-learning and deep-learning methods for optic disc (OD) and optic cup (OC). A total of 29 architectures were presented, and most works employed convolutional neural networks (CNN), U-Net-based approaches, and generative-adversarial network (GAN) approaches, and only one used region-based CNN (R-CNN), [8], in which the authors used a Faster R-CNN that transforms the predicted bounding box into a vertical and non-rotated ellipse. None of the reviewed models were on R-CNN for the segmentation task but one.

Besides standard models, other deep-learning-related architectures have been released. Examples of U-Net architectures reviewed can be consulted at [9,10,11,12], and segmentations based on GAN can be seen in [13,14,15,16]. Another recent work based on CNN is the one presented by B. Liu et al. [17]. The authors employed depthwise separable convolution layer and image pyramid input, with a dice score of 0.96 for OD and 0.89 for OC on the REFUGE dataset. Z. Tian et al., in [18], proposed a graph convolutional network that takes the feature map concatenated with the graph nodes as input for segmentation of OD and OC, achieving a dice index of 0.9776 and 0.9558, respectively. Y. Zheng et al., in [19], presented a multiscale CNN to generate initial contour and evolution parameters, reporting on the REFUGE dataset an intersection over union (IoU) of 0.9669 for OD and 0.9361 for OC. Finally, a novel approach in multicolor scanning laser image was employed by J. Zhang et al. in [20], in which the authors produced functional annotations through non-expert crowdsourcing, which takes advantage of several regularization networks and segmentation networks.

Object detection aims to count objects in a scene and determine and track their precise locations while accurately labeling them. These models are more robust to occlusions, challenging illumination, and complex scenes as an advantage.

In medical images, detecting objects of interest is fundamental to diagnosis, but unfortunately, it represents intensive work for clinicians. The work consists of the localization and identification of lesions or abnormalities in the whole image space. Object detection techniques could improve accuracy and decrease the reading time for human experts [21]. Because of that, various approaches have been proposed using object detection in medical image analysis for three main tasks: detection, classification, and segmentation, all of which are closely related to medical diagnosis. Object detection techniques can identify objects from an image, draw a bounding box around a region of interest, assign a class label, and segment an object. The object detection models have two approaches: one-stage object detectors and two-stage object detectors. Several works from both categories have been reviewed.

Some works apply object detection techniques, such as S. Sadhukhan et al. in [22], for OD localization, where they used a Faster R-CNN to achieve satisfactory accuracy and high speed compared to other works, and S. Ajitha et al. in [23], where they detected glaucoma through a Faster R-CNN. Authors in [24] proposed a Region Focus Network, where a novel multi-class mask branch was designed. In [25], P. Kakade et al. showed a detailed comparative study between four different deep learning algorithms: YOLOv2, YOLOv2 reduced, YOLOv3, and Mask RCNN, where in terms of accuracy, YOLOv3 showed the best performance; however, in terms of IoU, Mask R-CNN showed the best results. The authors in [26] proposed a Densenet-77 based Mask R-CNN to address blurred retinal images, reporting an IoU of 0.972. An essential domain-shift problem across different datasets was proposed by Y. Guo et al. in [27], throwing a coarse-to-fine adaptive Faster R-CNN for joint OD and OC segmentation. H. Almubarak et al. [28] made use of a simple two-stage Mask R-CNN, which first detects and cuts around the ONH, then introduces the cropped image with the original one into the new detection network using different scales. Finally, Z. Wang et al. [29], instead of detecting a bounding box, directly estimated the parameters of an ellipse that sufficed to capture the morphology of each OD and OC region for calculating the CDR.

Many techniques are used in the extensive state-of-the-art reviews on OD and OC segmentation, mainly those associated with CNNs, U-Net architectures, and GAN-based models. These models are trained and tested on various datasets that need to be augmented. On the other hand, object detection has not been widely addressed in this area, so a detailed analysis of the behavior of different object detection models is the motivation of this research, enriching the current literature while establishing the typical metrics of these models as a basis for future comparisons. Another reason is that these new models are traditionally tested on classic Common Objects in Context (COCO) [30] and The Pascal Visual Object Classes (PASCAL VOC) 2007 [31] datasets, and they will be employed over fundus retinal images, addressing the number of images needed to train deep neural networks, testing models on reduced- and full-dataset and stable comparisons. The statement of significance can be seen in Table 1.

After the introduction and a review of state-of-the-art practices, the article follows with the materials and methods in Section 2, where every model is described, including the framework utilized, annotation mechanics, and training configuration. Evaluation and results can be seen in Section 3, to report performance; discussion of results is found in Section 4, and conclusion of the work is seen in Section 5.

## 2. Materials and Methods

Currently, object detection models are being used for pose estimation, vehicle detection, and surveillance, among other applications. These algorithms try to draw a bounding box around the object of interest. It does not necessarily have to be one; it can be several different box dimensions and different objects.

MMDetection tools have been used in this investigation [32], which provides an integrated framework for object detection and instance segmentation based on Pytorch [33]. This tool belongs to the MMLab project, an open-source project for academic research and industrial applications. Significant features of MMDetection are modular design, support for multiple frameworks out-of-the-box, and high efficiency.

**Figure 1:**
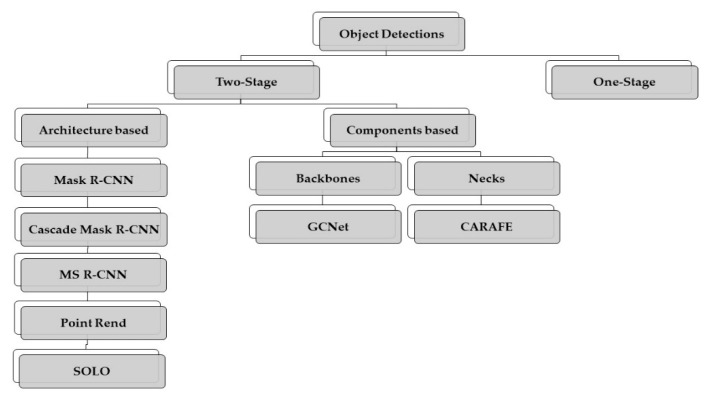
Object detection models used in this investigation.

The general intention of this study is to test various object detection models that can be useful in OD and OC segmentation, addressing the two-state categories of object detection models, which are generally more accurate than one-stage object detection. See Figure 1 for a presentation of the models that will be used. The two-stage framework comprises first a CNN for extracting features from original images and then a category-specific classifier for the class label.

Two approaches are taken to predict the presence of glaucoma with deep learning techniques. The first type of ONH assessment, choosing between healthy and glaucomatous, is an image-level task. However, although this task shows a high level of accuracy, a pixel-level job is preferred in this work, segmenting OD and OC, because this second type helps to obtain different signs used by doctors to predict the presence or absence of glaucoma.

### 2.1. Foundation in Object Detection with Deep Learning

Understanding critical elements of how these models work, why they are relatively slow but powerful, and the process of how sharing features improves the two-stage detector are the goals of this section.

#### 2.1.1. R-CNN

In [34], the first successful system for object localization, classification, and segmentation was created, taking from original images around 2000 of patched knows as a region. It then computes the feature for each proposal using a prominent CNN and finally classifies each region using class-specific linear-support-vector machines.

#### 2.1.2. Fast R-CNN

R-CNN is slow since each proposal region passes through a CNN without sharing computation. In more recent work [35], the entire image is passed through a CNN. It introduces ROI pooling as an input-to-output concatenation of the features extracted from each proposed region and fed into a fully connected layer during category prediction, with two outputs: one softmax probability and a per-class bounding-box regression offset.

#### 2.1.3. Faster R-CNN

Previous object-detection models still have a bottleneck with selective search, which has lethargic mechanics and time-consuming processes affecting the network’s performance. In [36], the concept of region-proposal networks was proposed: placed on top of the CNN features, it is then reshaped using RoI and classified, and both the bounding-box tasks are carried out.

#### 2.1.4. Mask R-CNN

Mask R-CNN [37] was introduced for predicting segmentation masks on each RoI with a small FCN on each one. This model extends Faster R-CNN, adding a new branch parallel to the existing branch for classifying and using the bounding box. This model slightly increases the computational cost but still is a fast system and allows rapid experimentation.

### 2.2. Common Components in Object Detection Architectures

A combination of ResNet50 [38], which overcame the degradation problem when models began to converge, and Feature Pyramid Network (FPN) [39], designed as an extractor of multiple levels of feature maps, was used as a backbone. The idea is to obtain semantic-rich layers from the bottom up and construct higher-resolution layers from the top-down side.

The bottom-up pathway starts with simple convolution over the input image. This first level is not included in the pyramid scheme because of extensive memory use. Then levels 2, 3, 4, and 5 use Resnet blocks with multiple convolution layers. The outputs of each level are reduced by a factor of 2, doubling the stride, and are the entry to the next level and used in the top-down pathway.

**Figure 2:**
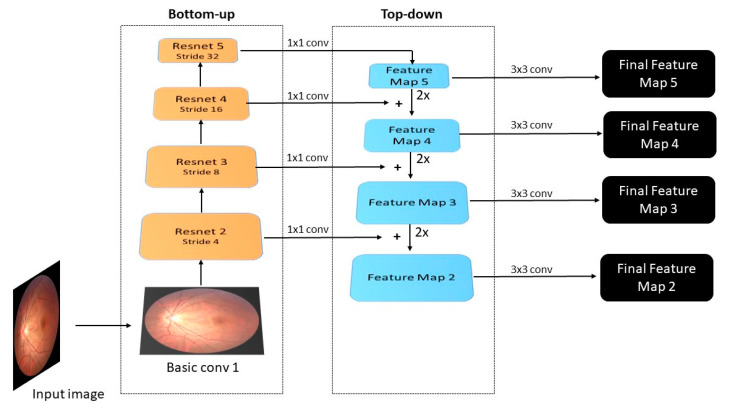
Detailed design of Resnet 50 + FPN backbone.

The top-down pathway begins with a 1 × 1 convolution filter to reduce channel dimensions to 256. The process is repeated at all levels, concatenating the same level output from the bottom-up side by element-wise addition. Each level is upsampled by two before addition. Finally, to obtain the final feature map at each level, a 3 × 3 convolution is applied. This helps with the superposition problem of upsampling. Figure 2 has a detailed schema of the backbone utilized.

Standard components in two-stage object-detection architectures are Backbone, Neck, DenseHead, RoIExtractor, and RoIHead.

- Backbone: The network takes an image as input and extracts the feature map without the last fully connected layer. The backbone can be a pre-trained neural network.
- Neck: Following the backbone, the neck layer extracts more elaborate feature maps from different stages.
- DenseHead: This works on dense locations of feature maps. An example is RPN, where anchor boxes are generated from anchor points founding it in feature maps. Scales and aspect ratios are crucial elements used to make candidate boxes.
- RoIExtractor: This component extracts RoI-wise features using RoI Pooling techniques and RoI Aling allowing transform non-uniform target cells to the same size.
- RoIHead: This takes RoI features into a specific task such as bounding-box classification/regression and mask prediction in instance segmentation.

### 2.3. Instance Segmentation Models

This investigation aims to extract the OD and OC as individual elements. The extraction task is related to instance segmentation, which allows the detection and localizing of an object in an image. The goal of instance segmentation is to allow objects of the same class to be divided into different instances; although, by concept, disc and cup are different classes, they are very alike in shape and overlap each other. For that reason, they need to be extracted separately. Object detection models can address this problem and will be covered here, assessing the performance of recent architectures.

#### 2.3.1. Cascade R-CNN

Cascade R-CNN is a multi-stage object-detection architecture where increased IoU thresholds are used in a sequence-order detector, using the output of one as a trainer of the next one, thereby improving quality, guaranteeing a positive training set, and minimizing overfitting [40]. This architecture is an extension of Faster R-CNN, and obtaining masks can be addressed in two ways: putting the segmentation branch at the beginning or end of Cascade R-CNN or in each stage. This last one maximizes the diversity of samples used to learn the mask-prediction task.

#### 2.3.2. Mask Scoring R-CNN

For most instance-segmentation models, the confidence of instance classification is used as a mask quality score. However, in practice, instance mask and ground truth usually are not well correlated with classification scores. The idea behind this architecture is to take the instance feature and the corresponding predicted mask together to regress the mask IoU throughout a MaskIoU head [41]. In this proposal, the predicted mask and ROI feature are taken as input to the MaskIoU head.

#### 2.3.3. PointRend: Image Segmentation as Rendering

This module brings us flexibility throughout point-based segmentation predictions at adaptively selected locations based on an iterative subdivision algorithm. The PointRend model provides crisp object edges in regions over smoothed by previous methods [42]. PointRend chooses a set of points to accomplish the task and predicts each point individually with a small multilayer perceptron, using interpolated features computed at these points. This process is applied sequentially to optimize conflicted regions of the predicted mask.

#### 2.3.4. CARAFE

Content-Aware ReAssembly of Features exploits a large field of view, aggregating contextual information. It enables instance-specific, content-aware handling, generating adaptive kernels instantly, and is lightweight and quick to compute [43]. Carafe is composed of two principal components: the kernel prediction module generates reassembly kernels in a content-aware manner, whereas the content-aware reassembly module reconstructs the features from each reassembly kernel within a local region with a specific function.

#### 2.3.5. GCNet

Global Context Network was proposed to enhance NLNet, which is tasked with capturing long-range dependencies via aggregating query-specific global contexts foreach query position. The improvement was set up with a three-step general framework, obtaining a better instantiation based on a query-independent formulation [44].

#### 2.3.6. SOLO

Segmenting Objects by Locations [45] introduces “instance categories,” an approach that assigns categories to each pixel within an instance according to its location and size; this approach transforms instance segmentation into a one-step classification problem. The proposed model divides the input image into a uniform grid, and if the center of an object falls into a grid cell, this one predicts the semantic category and segments that object instance.

**Figure 3:**
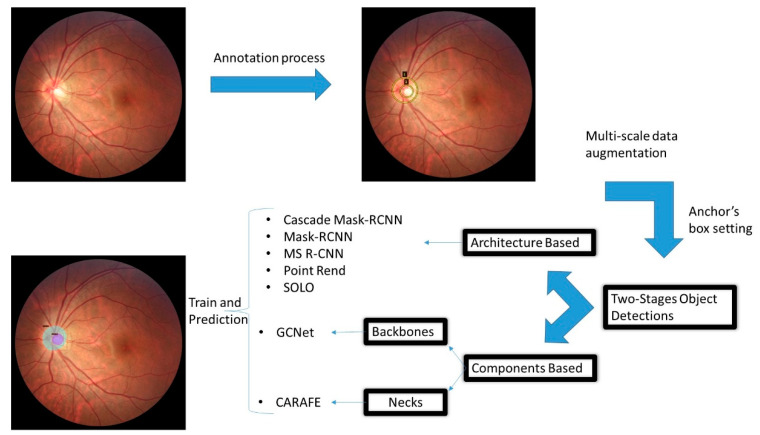
Workflow in the proposed investigation. In the annotation process, the ellipse with the number 1 is related to the disc class, and ellipse two is related to the cup class.

The reviewed models can be summarized in Figure 3, where the main contribution of this investigation is highlighted. The importance of this methodology is that it allows us to analyze the behavior of object detection models based on an architectural approach, backbone components, or components in the neck. The overall workflow starts with retrieving the images and annotating them accordingly. Then, we set up the experimentation with and without multiscale and appropriate anchor-box configuration before training and predicting the segmented area.

### 2.4. Materials

#### 2.4.1. Device and Databases

The device used in all experiments is a PC with an Intel(R) Core (TM) i5-8400 processor CPU@2.80 GHz, with 16 GB RAM and an NVIDIA GeForce GTX 1070 graphics card, with 8 GB VRAM. The software packages used were Python, in version 3.9.7, from the authors’ Van Rossum, Guido and Drake, in Scotts Valley, California, Pytorch in his version 1.10.0, developed by a laboratory of Facebook, today Meta, and is an open-source tool, MMDetection, version 2.19.0, and MMCV, version 1.4.0, which is a foundational library for computer vision and support projects such as MMDetection [46], all open source.

Two databases were used:

(a) REFUGE [47]: The Retinal Fundus Glaucoma Challenge was the first challenge on glaucoma assessment from retinal fundus photography and is one of the most extensive public datasets available for cup/disc segmentation. It consists of 1200 retinal images in JPEG format. Two devices were used: a Zeiss Visucam 500 fundus camera with a resolution of 2124 × 2056 pixels (400 images) and a Canon CR-2 with a resolution of 1634 × 1634 pixels (800 images). The macula and optic disc are visible in each image, centered at the posterior pole.
(b) G1020 [48]: A new public dataset for cup/disc segmentation and images was collected at a private clinical practice in Kaiserslautern, Germany, between 2005 and 2017, with a 45-degree field of view after dilation drops. Experts marked optic-disc and cup boundaries and bounding-box annotations using labelme [49], a free, open-source tool for annotations. Images are stored in JPG format with sizes between 1944 × 2108 and 2426 × 3007 pixels.

#### 2.4.2. Experimentation

Different model implementations provided by the MMDetection framework were used to operate experiments.

##### Annotations and Pre-Processing

This framework supports the COCO-style dataset and its large-scale object detection, segmentation, and captioning dataset. It is crucial to determine the precise location of optic and cup discs. VGG Image Annotator (VIA) [50] software was used to generate the annotations of the RoI for a proper and correct training procedure. This software is open source, and it is a standalone and straightforward manual-annotation platform for images, audio, and video. REFUGE dataset was set up through these tools and then exported in COCO format.

**Figure 4:**
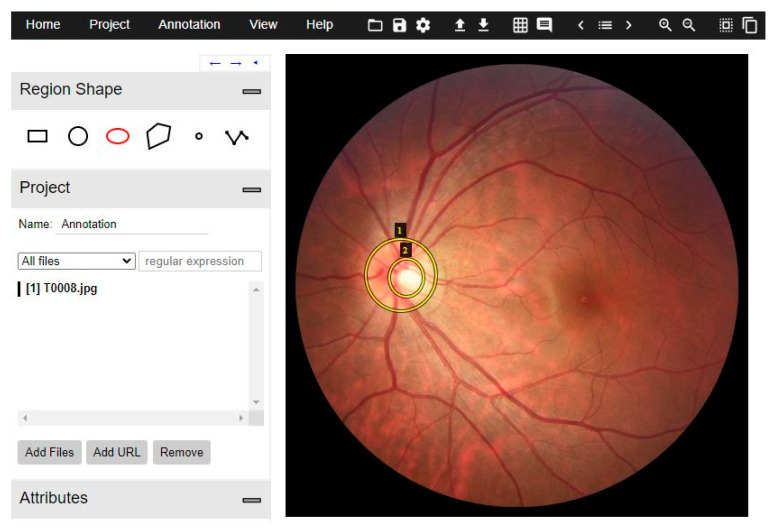
Annotation example of REFUGE image. The elliptic shape was selected. Number one annotates to disc class, and annotation two corresponds to cup class.

An example can be seen in Figure 4. An elliptical shape was selected since this is what best fits the optic and cup discs. The original ground truth of both datasets was used as a guide.

Pre-processing and augmenting images are always vital to a neural network’s successful behavior; however, aggressing transformations do not always lead to better results. In this work, a few steps were made. Resizing was done, adopting a simple data augmentation scheme based on multiscale training where images were taken with sizes between 1333 × 640 and 1333 × 960 with a step of 32 between each other; this approach shows high performance in terms of average precision (AP), bounding box, and masks with respect to one fixed size. Then, a random flip follows normalization based on the mean and standard deviation of ImageNet [51], commonly used as transfer learning to speed up the training process.

##### Training Setting

The training pipeline was set up following the original setting of the framework used in [32]. However, minor changes were made with a view to improving the performance. As an optimizer, AdamW was used [52], with an initial learning rate of 0.0025. The original framework worked with a value of 0.02 for 8 GPU; since here it worked with only one, this original value was divided by 8, and as a learning rate schedule, cosine annealing was utilized, allowing warm restart techniques to improve performance when training deep neural networks [53]. Cosine annealing was initially developed for the Stochastic Gradient Descend (SGD) optimizer, but new studies suggest a better performance when cosine annealing is used with AdamW [54].

The total loss of an object detector is the combination of classification, localization, and segmentation losses due to the multi-task nature of these models. Equation (1) shows the formula.

(1) $$L_{Total}=L_{Cls}+L_{Reg}+L_{Mask}$$

$L_{Cls}$ is the loss of classification and uses a log loss function over two classes, $p_{i}$, the predicted probability, and $q_{i}$, ground truth label, see Equation (2).

(2) $$L_{CLs}\left(p_{i},q_{i}\right)=-q_{i}logp_{i}-\left(1-q_{i}\right)\log\left(1-p_{i}\right)$$

$L_{Reg}$ is the loss of bounding box regression. The mean square error is typically applied between original points $u_{i}$ and predicted points $v_{i}$ over the center coordinate, width, and height vector, i∈[x, y, w, h], see Equation (3).

(3) $$L_{Reg}=MSE\left(u_{i},\;v_{i}\right)$$

Finally, $L_{Mask}$ employs an average binary cross-entropy function over a mask of dimension *m x m* associated with ground truth class *k*. See Equation (4) for details, where $x_{i,j}$ is the cell’s label (*i*, *j*) in the ground truth and $y_{i,j}^{k}$ is the generated value from the model of the same cell and class *k* [37].

(4) $$L_{Mask}=-\frac{1}{m^{2}}\underset{1\leq i,j\leq m}{\sum }x_{i,j}logy_{i,j}^{k}+\left(1-x_{i,j}\right)\log\left(1-y_{i,j}^{k}\right)$$

Another characteristic that has been tuned is anchor boxes, an essential parameter for quality object detection. Anchor settings are specified with anchor scales and ratios, while anchor strides correspond to feature-map strides. To obtain more scales in each location, to increase the possibility of fixing the object correctly, more scales and ratios were added.

## 3. Evaluations and Results

After setting up the different models, it is crucial to evaluate their performances. COCO evaluation metrics have been adopted [55], and, as the primary metric, AP is used, with 10 Intersection over Union (IoU) thresholds of 0.50:0.05:0.95. This approach rewards detectors with better localization. AP, a measure to summarize the precision-recall curve into a single value representing the average of all precisions at multiple recall levels, can be seen in Equation (5) [31].

(5) $$Average\;Precision=\sum_{i=0}^{i=n-1}\left(r_{i+1}-r_{i}\right)p_{interp}\left(r_{i+1}\right),$$

where *r* is the total number of relevant samples, *n* the numbers of threshold and $p_{interp}$ is the precision at each recall level *r*, defined by Equation (6), and where $p\left(\tilde{r}\right)$ is the measured precision at recall $\tilde{r}$, which is the recall that exceeds *r.*

(6) $$p_{interp}\left(r_{i+1}\right)=\max p\left(\tilde{r}\right),\;\tilde{r}\geq r_{i+1},$$

This equation is based on the following sub-metrics: IoU, recall, and precision. According to [56], these metrics are well suited for medical image segmentation, along with the F1 score or Dice similarity coefficient, which is slightly different from IoU because this one penalizes under- and over-segmentation more than the Dice does. The Dice coefficient is often used to quantify the performance of image segmentation methods. This metric is an order of how similar two objects are.

Their calculation is based on four possible interpretations of the data: True Positive (TP), True Negative (TN), False Positive (FP), and False Negative (FN). Based on these values, the following formulas can be defined for IoU, Recall, Precision and F1-score in Equations (7)–(10), respectively.

(7) $$IoU=\frac{TP}{TP+FP+FN},$$

(8) $$Recall=\frac{TP}{TP+FN},$$

(9) $$Precision=\frac{TP}{TP+FP},$$

(10) $$F1\;score=\frac{2\times Precision\times Recall}{Precision+Recall},$$

Experimentation starts with a fraction of the Refuge dataset, specifically 100 images for training, 30 for validation, and 30 for the test. This subset was taken, due to the cumbersome nature of the labeling process, to observe a first approximation of the behavior of the models to be evaluated. It also gives us an idea of how well the model behaves with a limited number of images. The task was developed without multiscale (WM) and multiscale (M).

Three criteria are reported, AP [IoU = 0.50:0.95], where AP is averaged over multiple IoU values, which rewards detectors with better localization. This will be taken as the primary metric. AP [IoU = 0.50] and AP [IoU = 0.75] are reported, as well, since they are more common in the literature and results are more tightened. Loose thresholds are reported as well. Three models improve their performance with multiscale: GCNet, MS-RCNN, and Point_Rend. The best result overall was Mask-RCNN with AP [IoU = 0.50:0.95] with 0.671. Results can be seen in Table 2.

After that, the experiment was repeated under the same conditions with the full Refuge dataset, using 400 images for training, 200 for validation, and 200 for testing. Results can be seen in Table 3.

Except for Carafe, all models improved their performance with the multiscale approach. The experiments were run on the G1020 dataset, with multiscale, since this approach shows better results. Results are shown in Table 4.

**Figure 5:**
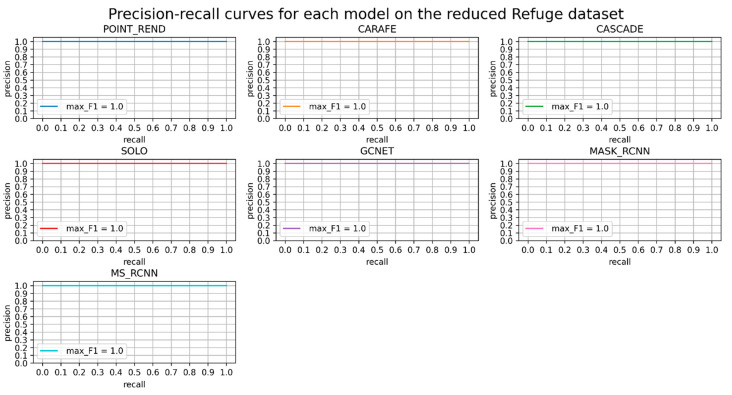
Precision-recall curve for each model on the reduced Refuge dataset. F1 score is provided for each model.

Precision-recall curves are provided for better understanding. The first plot, see Figure 5, shows that all models perform excellently, meaning that recall increases by some amount and precision is unchanged; thus, all retrieved are true positives.

**Figure 6:**
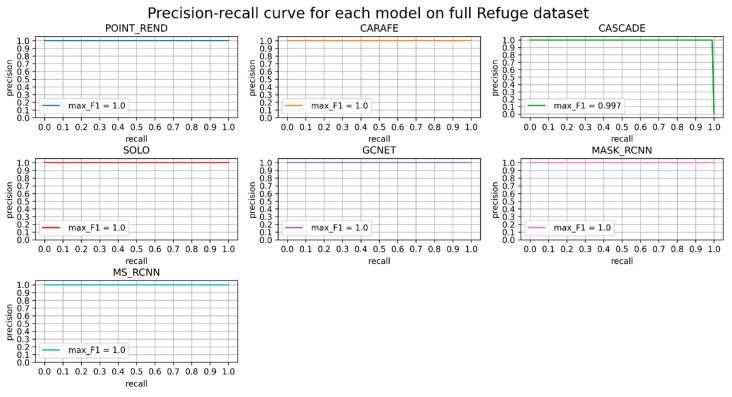
Precision-recall curve for each model on full Refuge dataset. F1 score is provided for each model.

In Figure 6, as in the previous one, all model performances are perfect except Cascade Mask-RCNN. This model cannot retrieve all true positives.

**Figure 7:**
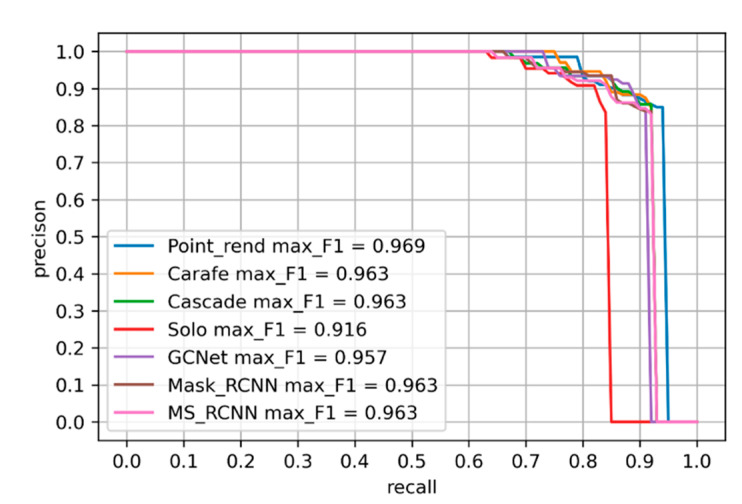
Precision-recall curves for each model on G1020 dataset. F1 score is provided for each model.

Figure 7 shows promising results as well. However, this plot implicitly shows the presence of false positives associated with localization errors, class confusion, and false negatives. These errors occur because the G1020 dataset presents high diversity in its images compared to the Refuge dataset.

**Figure 8:**
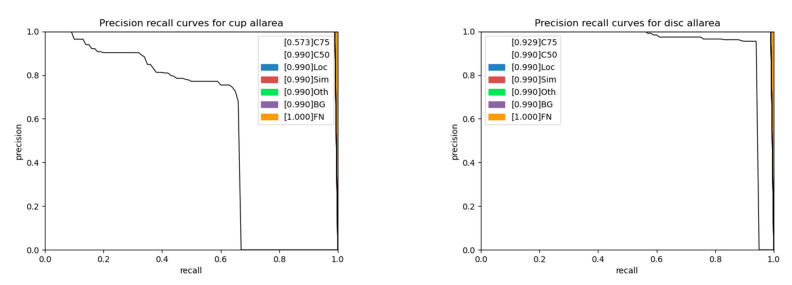
Precision-recall curves per classes in Cascade Mask-RCNN on Refuge dataset. The left image represents the cup area, and the right image the disc area. Both show the presence of false positives.

A series of precision-recall (PR) curves for each class are given for interpretation purposes in Figure 8. Each PR curve is guaranteed to be higher than the previous one as the evaluation setting becomes more permissive regarding the IoU threshold. The legend is described as follows, with the meaning of each curve [55].

1. C75: area under the curve corresponds to AP_[IoU=0.75]_ metric.
2. C50: area under the curve corresponds to AP_[IoU=0.50]_ metric.
3. Loc: localization errors are ignored, but not duplicate detections.
4. Sim: PR after supercategory false positives (fps) are removed.
5. Oth: PR after all class confusions are removed.
6. BG: PR after all background (and class confusion) fps are removed.
7. FN: PR after all remaining errors are removed (trivially AP = 1).

An explanation of Figure 6, where the precision-recall curve was not perfect for the Cascade Mask-RCNN model on the Refuge dataset, is presented by a per-class analysis. Figure 8 shows a thin line at the end of the plot exhibiting the presence of the false negative for each class.

**Figure 9:**
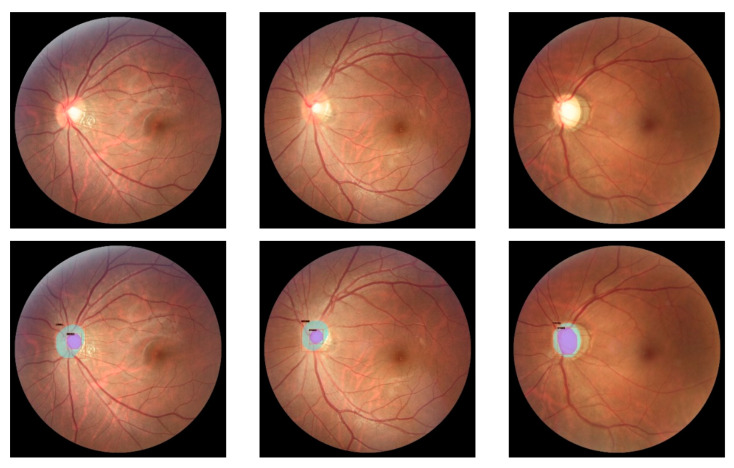
Output result from MS-RCNN on some images from test Refuge dataset. First row original images, second row segmented images.

**Figure 10:**
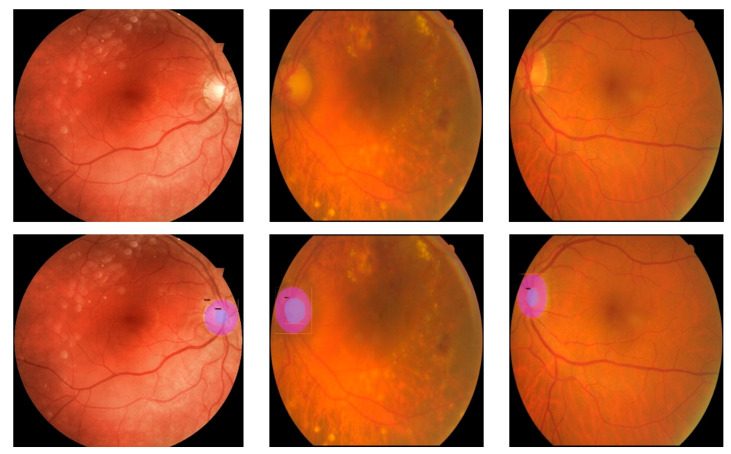
Output result from MS-RCNN on some images from test G1020 dataset. First row original images, second row segmented images.

In Figure 9 and Figure 10, segmentation results can be seen for MS-RCNN.

**Figure 11:**
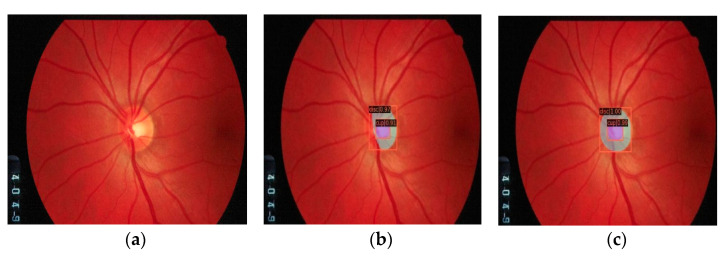
Segmentation on extern dataset with Cascade Mask-RCNN model. Original image from DRIONS-DB (**a**). Segmentation result from trained model with Refuge dataset (**b**). Segmentation result from trained model with G1020 dataset (**c**).

**Figure 12:**
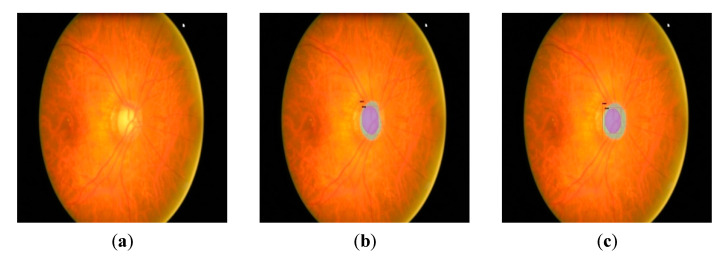
Segmentation on extern dataset with Cascade Mask-RCNN model. Original image from ORIGA-DB (**a**). Segmentation result from trained model with Refuge dataset (**b**). Segmentation result from trained model with G1020 dataset (**c**).

Models trained with the G1020 dataset from multiple experiments gave better results than the Refuge dataset when the trained models were applied to new datasets because of more diversity in their images. The test was performed on multiple images; for illustrative purposes, an image from DRIONS-DB [57] and ORIGA DB [58] are shown in Figure 11 and Figure 12, respectively, with the Cascade Mask R-CNN model.

As seen in the previous figure, the segmentation obtained for the OD does not cover the entire area expected with the models trained on the Refuge dataset, see Figure 11b. This result can be seen when the prediction is made with the model trained on the G1020 dataset, see Figure 11c. In the following Figure 12, the degradation of OD segmentation can be seen as well, comparing models trained on the Refuge and G1020 datasets.

## 4. Discussion

In this investigation, the objective was to compare different object detection models for segmenting the OD and OC on fundus images. Selected models were the original Mask R-CNN, Carafe, Cascade Mask R-CNN, GCNet, MS R-CNN, SOLO, and Point_Rend. The performance was evaluated based on average precision, F1-score, and area under the precision-recall curve (AUCPR).

The experimentation started with a small number of images to see the behavior of the model with a reduced number of images in obtaining a good segmentation of both OD and OC. According to [59], between 150–500 images there is a turning point where the performance of the models starts to increase significantly. We initially used 100 refuge images for training, 30 for validation, and 30 for testing. With this ratio, the best performances were associated with the Cascade, Mask R-CNN, and Point_Rend models with a value of 1.000 in average precision and an IoU threshold of 50. However, the rest of the models had good performances, around 0.98, except for SOLO, which resulted in a value of 0.886; this is because of a higher number of false positives due to incorrect detection of a non-existing object or a misplaced detection of an existing object. According to [60], false positives can be related to classification, localization, classification plus localization, duplication, and background errors. The slight decrease in SOLO may be associated with localization errors when an object is detected with a misaligned bounding box, meaning an overlap between 0.1 and 0.5 IoU. This problem can be addressed using negative samples in the training process [61,62]. Calculating the F1 score yielded a perfect value of 1.

With these previous results, we proceeded to annotate the entire Refuge data set and repeat the experimentation, where MS R-CNN outperformed the other models, with an average precision of 0.995 without using multiscale and 1.000 when multiscale was applied, always taking the IoU threshold equal to 50. This data augmentation technique slightly improved performance in all models except for the SOLO model, which decreased from 0.989 to 0.984, but this improved with the increase of images compared to the previous experimentation. Concerning the F1 score, all models maintained a perfect value of 1 except for Cascade Mask R-CNN, which decreased to 0.997. 

We experimented with these results on the G1020 dataset, as already noted previously, with the best performance being obtained by the Point_Rend model with an average precision value of 0.956. The remaining models maintained values around 0.94 except for the SOLO model, which decreased to 0.909. The decrease in the dataset may be related to more significant heterogeneity in its images; however, this resulted in an advantage at the time of transferring the predictions to images from other sources that were not used in the training process. Thus, the G1020 dataset was better at segmenting both the optical disc and optical cup than Refuge.

Key elements used in this research were multiscale, varying the values between 1333 × 640 to 1333 × 960. This approach introduced slight improvements in the metrics when the number of images increased, allowing the possibility of training models with new dimensions. However, this approach did not bring significant improvements with few images, so a researcher could consider its use, or not, depending on their resources.

Another element that was adjusted was the anchor scale. The configuration of the anchors is given by anchor_scales and anchor_ratios, and originally there were four generated in the region-proposal network. After using anchor_scales [4, 6, 8, 10, 12, 12, 14, 16] for anchor_ratios [0.5, 1.0, 1.5, 2.0], 28 anchors were generated for each feature- pyramid network level. This modification allowed us to trap a greater diversity in disc shapes and optical cups.

From the results, the perfect F1-score values are related to the precision-recall curves. The horizontal line means that all positives have been classified as positive (recall = 1), and all positives have been classified as true positives (precision = 1). G1020 shows excellent results as well, but these minor differences are because of higher variability in the images in this dataset.

## 5. Conclusions

Segmenting tasks are complex and time consuming in general. Traditional approaches use an encoder–decoder design, but this work has explored models based on seven object-detection algorithms for OD and OC segmentation, chosen from the many new models that have been released.

This investigation can set a precedent for comparing object detection models for localization, classification, and segmentation of OD and OC. Refuge and G1020 datasets were used, and a simple but effective mechanism for annotating input images was demonstrated over the first dataset. A fraction was initially taken from the Refuge dataset and evaluated with great results, proving that based on the result obtained, 100 images could be a good start, providing excellent results and decreasing the cumbersome process of annotating the images, but working with all datasets improved the average precision by around 0.050%. Another common problem addressed here was image reduction. Most studies of state-of-the-art procedures reduce input images significantly or introduce a new workflow in the segmentation process by cropping the area of interest. This work, with data augmentation based on multiscale, is proven to enhance results, keeping high-resolution input images and avoiding significant reductions. Anchor boxes setting for regional proposals improve target location too. AdamW optimizer and cosine-annealing strategy in the learning-rate schedule also slightly improved.

However, some limitations were identified in this research, such as the need for annotated images, which remains a substantial obstacle in the training of object detection models. The annotation process by specialists is full of variability that can be related to both human factors and image quality. Due to the complexity of evaluating segmentations, it is difficult to establish comparisons and select the best architectures since different metrics are used by researchers. Additionally, researchers use different data sources, so that even under the same training and testing conditions, it becomes impossible to compare two works if the same datasets were not used. Another limitation was the impossibility of training all the models with the different backbones that can be used, due to computational capacity and the time-consuming task of testing all the variants.

In general, all models perform almost perfectly in Refuge, while in G1020 high values of the F1 score were reached too. However, M. N. Bajwa et al. in [48] showed a higher F1 score, but they segmented the cup and the disc separately, making the task less challenging, while in this investigation they were processed together.

Deeper networks such as Resnet101 and ResnetX [32] can extract more features because of the number of layers; however, Resnet50 gives almost perfect results, being the least complex model, extracting correct features. Selecting the correct model depends upon hardware capability and time available since more extensive backbones imply a long processing time.

Future work is to extend the analysis to tiny objects and multi-class targets, especially crowded objects in retina images.

## Figures and Tables

**Table 1:** Statement of significance of this investigation.

**Problem**	Interpreting the segmentation of the OD and OC is crucial in diagnosing glaucoma. Accurate results can make the difference between a good and a poor prediction.
**What is Already Known**	Deep neural networks are used for segmentation, focusing mainly on encoder–decoder models. Heavy pre-processing and post-processing work. Pipelines are based on previously extracting the region of interest to perform the segmentation on that trimmed area.
**What This Paper Adds**	Evaluate state-of-the-art new object detection models with a two-stage approach, highlighting the best average precision. These models unified the detection and segmentation task. Addressed the traditional question of how many images are needed to train a deep-neural-network model. Experimentation performance into a subset and full dataset. The effect of multiscale data augmentation technique and the importance of a correct configuration of anchor’s scale for object location.

**Table 2:** Average precision results on reduced Refuge dataset.

Model Architecture	AP [IoU = 0.50:0.95]		AP [IoU = 0.5]		AP [IoU = 0.75]	
	WM	M	WM	M	WM	M
CARAFE	0.657	0.607	0.979	0.965	**0.771**	0.621
Cascade Mask-RCNN	0.618	0.608	**1.000**	**0.980**	0.661	0.646
SOLO	0.555	0.530	0.886	0.886	0.613	0.586
GCNET	0.584	0.595	0.980	0.960	0.608	0.638
MASK-RCNN	**0.671**	0.616	**1.000**	0.962	0.743	0.635
MS-RCNN	0.604	**0.627**	0.980	0.978	0.649	**0.676**
POINT_REND	0.582	0.607	**1.000**	0.965	0.564	0.621

**Table 3:** Average precision results on full Refuge dataset.

Model Architecture	AP [IoU = 0.50:0.95]		AP [IoU = 0.50]		AP [IoU = 0.75]		F1-Score
	WM	M	WM	M	WM	M	
CARAFE	0.650	0.636	0.990	0.995	0.710	0.685	**1.0**
Cascade Mask-RCNN	0.644	**0.661**	0.985	0.990	0.716	**0.739**	0.997
SOLO	0.610	0.647	0.989	0.984	0.676	0.703	**1.0**
GCNET	0.631	0.656	0.990	0.995	0.712	0.729	**1.0**
MASK-RCNN	0.595	0.629	0.948	0.988	0.662	0.701	**1.0**
MS-RCNN	**0.654**	0.658	**0.995**	**1.000**	**0.766**	0.738	**1.0**
POINT_REND	0.632	**0.661**	0.990	0.994	0.670	0.735	**1.0**

**Table 4:** Average precision results on G1020 dataset.

Model Architecture	AP [IoU = 0.50:0.95]	AP [IoU = 0.50]	AP [IoU = 0.75]	F1-Score
	M	M	M	
CARAFE	0.624	0.948	0.632	0.963
Cascade Mask-RCNN	0.631	0.947	0.662	0.963
SOLO	0.568	0.909	0.583	0.916
GCNET	0.628	0.943	0.646	0.957
MASK-RCNN	0.613	0.941	0.621	0.963
MS-RCNN	**0.638**	0.944	**0.664**	0.963
POINT_REND	0.617	**0.956**	0.648	**0.969**
